# A linear time algorithm for linearizing quadratic and higher-order shortest path problems

**DOI:** 10.1007/s10107-024-02086-z

**Published:** 2024-05-09

**Authors:** Eranda Çela, Bettina Klinz, Stefan Lendl, Gerhard J. Woeginger, Lasse Wulf

**Affiliations:** 1 Institute of Discrete Mathematics, Graz University of Technology, Graz, Austria; 2 Institut of Operations and Information Systems, University of Graz, Graz, Austria; 3 Department of Computer Science, RWTH Aachen, Aachen, Germany

**Keywords:** Quadratic shortest path problem, Higher-order shortest path problem, Linearization, 90C20 Quadratic programming, 90C27 Combinatorial optimization, 68Q25 Analysis of algorithms and problem complexity, 90C35 Programming involving graphs or networks

## Abstract

An instance of the NP-hard Quadratic Shortest Path Problem (QSPP) is called linearizable iff it is equivalent to an instance of the classic Shortest Path Problem (SPP) on the same input digraph. The linearization problem for the QSPP (LinQSPP) decides whether a given QSPP instance is linearizable and determines the corresponding SPP instance in the positive case. We provide a novel linear time algorithm for the LinQSPP on acyclic digraphs which runs considerably faster than the previously best algorithm. The algorithm is based on a new insight revealing that the linearizability of the QSPP for acyclic digraphs can be seen as a local property. Our approach extends to the more general higher-order shortest path problem.

## Introduction

In this paper^1^ we consider the linearization problem for nonlinear generalizations of the *Shortest Path Problem (SPP)*, a classic combinatorial optimization problem. An instance of the SPP consists of a digraph $G = (V, A)$, a source vertex $s \in V$, a sink vertex $t \in V$, and a cost function $c: A\rightarrow \mathbb {R}$, which maps each arc $a\in A$ to its cost *c*(*a*). The cost of a simple directed *s*-*t*-path *P*, is given by^2^

1 $$\begin{aligned} \text {SPP}(P,c):=\sum _{a\in P} c(a)\, . \end{aligned}$$

The goal is to find a simple directed *s*-*t*-path in *G* which minimizes the objective (1). In general it is assumed that there are no circuits of negative weight in *G*.

Consider now a number $d\in \mathbb {N}$. The *Order-d Shortest Path Problem (SPP*_d_*)* takes as input a digraph $G = (V, A)$, a source vertex $s \in V$, a sink vertex $t \in V$, and an order-*d* arc interaction cost function $q_d: \{{B \subseteq A: |B| \le d}\} \rightarrow \mathbb {R}$. Thus $q_d$ assigns a weight to every subset of arcs of cardinality at most *d*. The cost of a simple directed *s*-*t*-path *P* is given by

2 $$\begin{aligned} \text {SPP}_d(P,q_d):=\sum _{S\subseteq P : |S|\le d} q_d(S)\, . \end{aligned}$$

The goal is to find a simple directed *s*-*t*-path in *G* which minimizes the objective function (2). For $d=2$ we obtain the *Quadratic Shortest Path Problem (QSPP)* which has already been studied in the literature [3, 11, 12, 20].

The QSPP arises in network optimization problems where costs are associated with both single arcs and pairs of arcs. This includes variants of stochastic and time-dependent route planning problems [17, 22, 23] and network design problems [10, 16]. For an overview on applications of the QSPP see [12, 20]. We are not aware of any publications for the case $d>2$.

While the SPP can be solved in polynomial time, the QSPP is an NP-hard problem, even for the special case of the adjacent QSPP where the costs of all pairs of non-consecutive arcs are zero [20]. The QSPP is an extremely difficult problem also from the practical point of view. Hu and Sotirov [12] report that a state-of-the-art quadratic solver can solve QSPP instances with up to 365 arcs, while their tailor-made B &B algorithm can solve instances with up to 1300 arcs to optimality within one hour. In contrast, instances of the SPP can be solved in a fraction of a second for graphs with millions of vertices and arcs.

Given the hardness of the QSPP, a research line on this problem has focussed on polynomially solvable special cases which arise if the input graph and/or the cost coefficients have certain specific properties. Rostami et al. [21] have presented a polynomial time algorithm for the adjacent QSPP in acyclic digraphs and in series–parallel graphs. Hu and Sotirov [11] have shown that the QSPP can be solved in polynomial time if the quadratic costs build a nonnegative symmetric product matrix, or if the quadratic costs build a sum matrix and all *s*-*t*-paths in *G* have the same number of arcs. These two polynomially solvable special cases of the QSPP belong to the larger class of the *linearizable* $\text {SPP}_d$ *instances*. Loosely speaking a $\text {SPP}_d$ instance is linearizable if there exists a linear cost function *c* (a so called *linearizing* cost function) such that replacing the old cost function with the new linear cost function *c* does not alter the cost of any source-sink-path, i.e. $\text {SPP}_d(P,q_d) = \text {SPP}(P,c)$ holds for any such path. A precise definition is given in Sect. 2.

The recognition of linearizable QSPP (SPP_d_) instances arises as a natural question. In this problem the task consists of deciding whether a given instance of the QSPP (SPP_d_) is linearizable and in finding the linearizing cost function in the positive case. The notion of linearizable special cases of hard combinatorial optimization problems goes back to Bookhold [1] who introduced it for the quadratic assignment problem (QAP). For symmetric linearizable QAP instances a full characterization has been obtained while only partial results are available for the linearizability of the general QAP, see [2, 7–9, 14, 18, 25]. The linearization problem has been studied for several other quadratic combinatorial optimization problems, see [5, 24] for the quadratic minimum spanning tree problem, [19] for the quadratic TSP, [6] for the quadratic cycle cover problem and [13] for general binary quadratic programs. Linearizable instances of a quadratic problem can be used to generate lower bounds needed in B &B algorithms. For example, Hu and Sotirov introduce the family of the so-called *linearization-based bounds* [13] for the binary quadratic problem. Each specific bound of this family is based on a set of linearizable instances of the problem. The authors show that well-known bounds from the literature are special cases of the newly introduced bounds. Clearly, fast algorithms for the linearization problem are important in this context.

While the linearization for the SPP_d_, $d\ge 3$, has not been investigated in the literature so far (to the best of our knowledge), the LinQSPP has been subject of investigation in some recent papers. In Çela et al. [3] proved that it is coNP-complete to decide whether a QSPP instance on an input graph containing a directed cycle is linearizable. Thus, a nice characterization of linearizable QSPP instances for such graphs seems to be unlikely. In the acyclic case, Hu and Sotirov first described a polynomial-time algorithm for the LinQSPP on directed two-dimensional grid graphs [11]. Recently, in [13] they generalized this result to all acyclic digraphs and proposed an algorithm which solves the problem in $\mathcal {O}(nm^3)$, where *n* and *m* denote the number of vertices and arcs in *G*.

Finally, let us mention a related concept, the so-called universal linearizability, studied in [3, 11]. A digraph *G* together with a fixed choice of source *s* and sink *t* is called *universally linearizable with respect to the QSPP* iff every instance of the QSPP on the input graph *G* is linearizable for every choice of the cost function *q*. In [11] it is shown that a particular class of grid graphs is universally linearizable. In [3] a characterization of universally linearizable grid graphs in terms of structural properties of the set of *s*-*t*-paths is given. Moreoever, for acyclic digraphs a forbidden subgraphs characterization of the universal linearizability is given in [3].

*Contribution and organization of the paper* In this paper we provide a novel and simple characterization of linearizable QSPP instances on acyclic digraphs. Our characterization shows that the linearizability can be seen as a *local* property. In particular, we show that an instance of the QSPP on an acyclic digraph *G* is linearizable if and only if each subinstance obtained by considering a subdigraph of *G* consisting of two *s*-*t*-paths in *G* is linearizable. Our simple characterization also works for the SPP_d_ and even for completely arbitrary cost functions, which assign some cost *f*(*P*) to every *s*-*t*-path *P* without any further restrictions. The latter problem is referred to as the *Generic Shortest Path Problem* (GSPP) and is formally introduced in Sect. 2. Indeed, the characterization of the linearizable instances of the SPP_d_ follows from the characterization of the linearizable instances of the GSPP, both on acyclic digraphs. We remark that in parallel and independent to our work, Matuschke proved an equivalent result [15, Theorem 6].

Further, we propose a linear time algorithm which can check the local condition mentioned above for the QSPP and the SPP_d_. We note that this is not straightforward, because the number of the subinstances for which the condition needs to be checked is in general exponential. As a side result our approach reveals an interesting connection between the LinQSPP and the problem of deciding whether all *s*-*t*-paths in a digraph have the same length. As a result, we obtain an algorithm which solves the LinQSPP in $\mathcal {O}(m^2)$ time, thus improving the best previously known running time of $\mathcal {O}(nm^3)$ obtained in [13]. Our approach yields an $\mathcal {O}(d^2 m^d)$ time algorithm for the LinSPP_d_ on acyclic graphs, thus providing the first (polynomial time) algorithm for this problem. Note that the running time of the proposed algorithms is linear in the input size for both problems, LinQSPP and LinSPP_d_, respectively. (The costs of all $\Omega (m^2)$ pairs of arcs, in the case of the QSPP, and the costs of all $\Omega (m^d/d!)$ subsets of arcs of cardinality *d*, in the case of the SPP_d_, need to be encoded in the input.)

Finally, we also obtain a polynomial time algorithm that given an acyclic digraph *G* computes a basis of the subspace of all linearizable order-*d* cost functions on *G*. Such a basis can be used to obtain better linearization-based bounds usable in B &B algorithms.

The paper is organized as follows. After introducing some notations and preliminaries in Sect. 2 we present the result on the characterization of the linearizable QSPP and SPP_d_ instances on acyclic input digraphs in Sect. 3. The algorithms for the linearization problems LinQSPP and LinSPP_d_ are presented in Sect. 4. Section 5 deals with computing a basis of the subspace of all linearizable order-*d* cost functions on an acyclic digraph *G*.

## Notations and preliminaries

The following notations related to paths in digraphs will be used throughout the paper. Given a digraph $G=(V,A)$, a simple directed *s*-*t*-path *P* in *G* is specified as a sequence of arcs $P=(a_1,a_2,\ldots ,a_p)$ such that $a_1$ starts at *s*, $a_p$ ends at *t*, non-consecutive arcs do not share a vertex and the end vertex of $a_{i}$ coincides with the start vertex of $a_{i+1}$ for any $i\in \{1,\ldots ,p-1\}$. The number *p* of arcs in *P* is called the length of the path. We sometimes use the same notation for a path *P* and the set of its arcs. We consider a single arc (*x*, *y*) as an *x*-*y*-path of length 1 and a single vertex *x* as a trivial *x*-*x*-path of length 0. Given an *x*-*y*-path $P_1$ and a *y*-*z*-path $P_2$, we denote the *concatenation* of $P_1$ and $P_2$ by $P_1 \cdot P_2$. We also consider concatenations of paths and arcs, that is, terms of the form $P \cdot a$ for some *x*-*y*-path *P* and some arc $a = (y, z)$.

### Definition 1

An instance of the SPP_d_ with an input digraph $G=(V,A)$, a source node *s*, a sink node *t* and a cost function $q_d$ is called linearizable if there exists a cost function $c: A\rightarrow \mathbb {R}$ such that for any simple directed *s*-*t*-path *P* in *G* the equality $\text {SPP}(P,c) = \text {SPP}_d(P,q_d)$ holds.

A linearizable instance of the QSPP is a linearizable instance of the $\text {SPP}_2$.

In the linearization problem, we are concerned with digraphs $G =(V, A)$ with a source vertex *s* and a sink vertex *t*. We denote by $\mathcal {P}_{st}$ the set of all simple directed *s*-*t*-paths.

We often assume that *G* is $\mathcal {P}_{st}$-*covered*, that is, every arc in *G* is traversed by at least one path in $\mathcal {P}_{st}$. Note that we can make this assumption without loss of generality for acyclic graphs: If some arc is not traversed by at least one *s*-*t*-path (which can be decided in linear time for acyclic graphs), then it has no effect on the linearizability of the instance, and so we can delete that arc.

Let $d \ge 2$ be a natural number. The *Order*-*d* *interaction costs* are given by a mapping $q_d: \{{B \subseteq A: |B| \le d}\}\rightarrow \mathbb {R}$, assigning a (potentially negative) interaction cost to every subset of at most *d* arcs. The cost $\text {SPP}_d(P,q_d)$ of some path *P* under interaction costs $q_d$ is defined as in equation (2). If *d* is unambiguously clear from the context, we use the more compact notation $f_q(P):=\text {SPP}_d(P,q_d)$. In this paper we explicitly allow the case $q(\emptyset ) \ne 0$, because this simplifies the calculations. The *linearization problem for the Order-d Shortest Path Problem* (LinSPP_d_) is formally defined as follows.
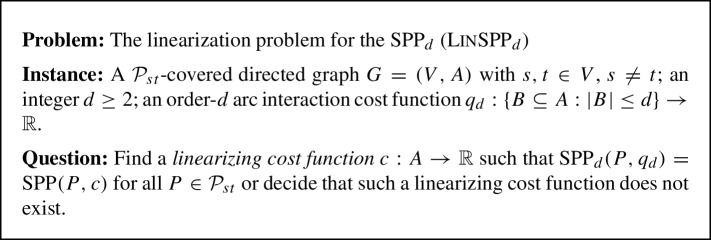


In the special case $d = 2$, we obtain the linearization problem for the QSPP (LinQSPP).

Finally, let us consider the *Generic Shortest Path Problem* (GSPP) which takes as input a digraph $G=(V,A)$ with a source vertex *s*, a sink vertex *t*, $s\ne t$, and a generic cost function $f: \mathcal {P}_{st}\rightarrow \mathbb {R}$ assigning a cost *f*(*P*) to every path $P\in \mathcal {P}_{st}$.^3^ The goal is to find an *s*-*t*-path which minimizes the objective function *f*(*P*) over $\mathcal {P}_{st}$. A linearizable instance of the GSPP and the linearization problem for the GSPP (LinGSPP) are defined analogously as in the respective definitions for SPP_d_.

We note that in our Definition 1 we allow the linearizing function $c: A \rightarrow \mathbb {R}$ to take negative values. For graphs with negative cycles, this could create an issue. Cycles are excluded however in acyclic graphs to which the results in this paper apply to. Note that for acyclic graphs the decicion problem about the existence of a nonnegative linearizing function can be reduced to the decision problem about the existence of a real-valued linearizing function, as shown by the following lemma.

### Lemma 1

If (*G*, *s*, *t*, *f*) is an instance of the GSPP such that $G = (V,A)$ is an acyclic digraph, then there is a nonnegative linearizing function $c': A \rightarrow \mathbb {R}_+$ if and only if $f(P) \ge 0$ for all *s*-*t*-paths *P* and there is a linearizing function $c: A \rightarrow \mathbb {R}$.

### Proof

If there is no linearizing function $c: A \rightarrow \mathbb {R}$, then there is also no linearizing function $c': A \rightarrow \mathbb {R}_+$.

Assume now that there exists a linearizing function $c: A \rightarrow \mathbb {R}$. Clearly, if there is an *s*-*t*-path *P* with $f(P) < 0$, then no nonnegative linearizing function exists. If $f(P) \ge 0$ for all *s*-*t*-paths, a nonnegative linearizing function $c'$ can be constructed by using the reduced costs $c_\pi (u,v):= \pi (u) - \pi ({v}) + c(u,v)$, for $(u,v) \in A$, where $\pi (v)$ denotes the cost of a shortest *s*-*v*-path with respect to *c*, for $v\in V$. We set $c'(a):= c_\pi (a) + \pi (t)$ for all arcs incident to the source and $c'(a):= c_\pi (a)$ otherwise. Since $\pi (t) \ge 0$ (due to the assumption that $f(P)\ge 0$ for all *s*-*t*-paths) and $c_\pi (u,v)\ge 0$, for all $(u,v) \in A$ (due to the acyclicity of *G*), $c'$ takes only nonnegative values. Moreoever, for all *s*-*t*-paths *P* the equalities $c_\pi (P) = c(P) + \pi (s) - \pi (t) = c(P) - \pi (t)$ and $c'(P)=c_\pi (P)+\pi (t)$ hold, implying $c'(P)=c(P)=f(P)$. $\square$

## A characterization of linearizable instances of the GSPP on acyclic digraphs

The main result of this section is Theorem 1, our novel characterization of linearizable instances of the GSPP on acyclic digraphs defined as in Sect. 2.

### Definition 2

Let $G=(V,A)$ be a $\mathcal {P}_{st}$-covered acyclic digraph. For some vertex *v*, let $P_1, P_2$ be two *s*-*v*-paths, and let $Q_1, Q_2$ be two *v*-*t*-paths. The 5-tuple $(v,P_1,P_2,Q_1,Q_2)$ is called a *two-path system* contained in *G*. The system is called *linearizable* with respect to the function $f: \mathcal {P}_{st}\rightarrow \mathbb {R}$, if there exists a cost function $c: A \rightarrow \mathbb {R}$ such that for all four paths $P \in \{{P_1 \cdot Q_1, P_1 \cdot Q_2, P_2 \cdot Q_1, P_2 \cdot Q_2}\}$ the equality $f(P) = SPP(P,c)$ holds. Such a *c* is called a *linearizing cost function* for $(v,P_1,P_2,Q_1,Q_2)$ with respect to *f*.

**Fig. 1:**
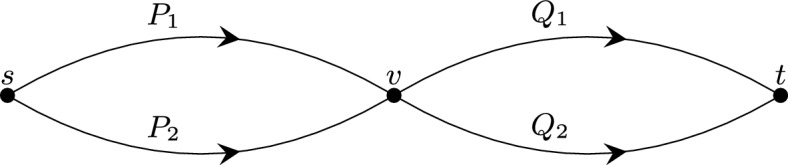
A two-path system

See Fig. 1 for an illustration of a two-path system. Note that $P_1$ and $P_2$ (as well as $Q_1$ and $Q_2$) can have common inner vertices and that the cases $P_1=P_2$, $Q_1=Q_2$, $v = s$ and $v = t$ are allowed. However, due to the acyclicity of *G*, the paths $P_i$ and $Q_j$ have only the vertex *v* in common for $i,j \in \{1,2\}$. Further, observe that the linearizability of a two-path system is a local property, in the sense that it only depends on the four paths $P_1 \cdot Q_1, P_1 \cdot Q_2, P_2 \cdot Q_1$ and $P_2 \cdot Q_2$. Indeed, the following simple characterization holds.

### Proposition 1

A two-path system $(v,P_1,P_2,Q_1,Q_2)$ is linearizable with respect to some function $f: \mathcal {P}_{st}\rightarrow \mathbb {R}$ iff

3 $$\begin{aligned} f(P_1 \cdot Q_1) + f(P_2 \cdot Q_2) = f(P_1 \cdot Q_2) + f(P_2 \cdot Q_1). \end{aligned}$$

### Proof

First, assume that $(v,P_1,P_2,Q_1,Q_2)$ is linearizable and let *c* be the corresponding linearizing cost function. Let $M_1$ ($M_2$) be the multiset resulting from the union of the sets of the arcs of the paths $P_1\cdot Q_1$ and $P_2\cdot Q_2$ ($P_1\cdot Q_2$ and $P_2 \cdot Q_1$). Since $M_1$ and $M_2$ coincide we get $c(P_1 \cdot Q_1) + c(P_2 \cdot Q_2) =\sum _{a\in M_1} c(a)= \sum _{a\in M_2} c(a)=c(P_1 \cdot Q_2) + c(P_2 \cdot Q_1)$. Then, (3) follows from the definition of the linearizability of $(v,P_1,P_2,Q_1,Q_2)$.

Assume now that Equation (3) is true. We show the linearizability of the two-path system with respect to *f* by constructing a linearizing cost function *c*. It is easy to find a suitable *c* if $P_1=P_2$ or $Q_1=Q_2$. (Indeed, if both $P_1 = P_2$ and $Q_1 = Q_2$, then the four paths collapse to a single path, which is clearly linearizable. If exactly one of the two equations $P_1 = P_2$ and $Q_1 = Q_2$ holds, then the four paths collapse to two paths. The two paths together can be linearized by giving appropriate costs to the arcs unique to each path.)

So let us consider the more general case where $P_1\ne P_2$ and $Q_1\ne Q_2$. In this case, for each $P\in \{P_1,P_2,Q_1,Q_2\}$ there exists a (not necessarily unique) *representative arc* $a\in P$ such that *a* is not contained in any other path $Q\in \{P_1,P_2,Q_1,Q_2\}$, $Q \ne P$. Let $a_1$, $a_2$, $e_1$, $e_2$ be the representative arcs of $P_1$, $P_2$, $Q_1$ and $Q_2$, respectively. Consider now a cost function $c: A \rightarrow \mathbb {R}$, such that $c(a) = 0$ if $a\not \in \{a_1, a_2, e_1, e_2\}$, and $c(a_1)$, $c(a_2)$, $c(e_1)$, $c(e_2)$ fulfill the following linear equations:

$$\begin{aligned} \begin{array}{llcllcl} c(a_1) + c(e_1) = f(P_1 \cdot Q_1) \\ c(a_1) + c(e_2) = f(P_1 \cdot Q_2) \\ c(a_2) + c(e_1) = f(P_2 \cdot Q_1) \\ c(a_2) + c(e_2) = f(P_2 \cdot Q_2) \end{array} \end{aligned}$$

Using basic linear algebra, one can see that this system indeed has a solution whenever Equation (3) holds (there is even a solution with $c(e_2) = 0$). Thus, *c* constructed as above is a linearizing cost function for $(v,P_1,P_2,Q_1,Q_2)$ with respect to *f*. $\square$

Now, consider an instance of the GSPP with a $\mathcal {P}_{st}$-covered acyclic digraph *G*, with a source vertex *s*, a sink vertex *t* and a generic cost function $f: \mathcal {P}_{st}\rightarrow \mathbb {R}$. When is this instance (*G*, *s*, *t*, *f*) linearizable? Obviously, if *G* contains a two-path system which is not linearizable with respect to *f*, then the instance (*G*, *s*, *t*, *f*) as a whole is also not linearizable. Interestingly, this necessary condition turns out to be also sufficient.

### Theorem 1

Let *G* be a $\mathcal {P}_{st}$-covered acyclic digraph with a source vertex *s* and a sink vertex *t* and let $f: \mathcal {P}_{st}\rightarrow \mathbb {R}$ be a generic cost function. Then the instance (*G*, *s*, *t*, *f*) of the GSPP is linearizable if and only if every two-path system contained in *G* is linearizable with respect to *f*.

Before proving the theorem, we need some preparation. Let $G = (V,A)$ be a $\mathcal {P}_{st}$-covered acyclic digraph with source vertex *s* and sink vertex *t*.

We use the concept of a *topological arc order* defined as a total order $\preceq$ on *A* which has the following property: for any pair of arcs *a*, $a'$ in *A*, if there exists a path *P* containing both *a* and $a'$ such that *a* comes before $a'$ in *P*, then $a\preceq a'$.

It is easy to see that any acyclic digraph has a (in general non-unique) topological arc order. Moreover, a topological arc order can be obtained from a topological vertex order.

Further, we recall the definition of a *system of nonbasic arcs* introduced by Sotirov and Hu [13].

### Definition 3

Let *G* be a $\mathcal {P}_{st}$-covered acyclic digraph with a source vertex *s* and a sink vertex *t*. A set $N\subseteq A$ is called a *system of nonbasic arcs*, iff for every vertex $v \in V \setminus \{{s,t}\}$ exactly one of the arcs starting at *v* is contained in $N$. The latter arc is called the *nonbasic arc of* *v*. An arc $a \in A \setminus N$ is called *basic*.

Obviously, the system of nonbasic arcs is not unique. Any such system forms an in-tree rooted at *t* containing all the vertices in *V* except for *s*. For some system of nonbasic arcs $N$ and some vertex $v \in V \setminus \{{s}\}$, we let $N_v$ denote the unique *v*-*t*-path consisting of nonbasic arcs (where $N_t$ is the trivial path). A cost function $c: A \rightarrow \mathbb {R}$ is called *in reduced form* with respect to $N$, if $c(a) = 0$ for all nonbasic arcs $a \in N$. The following lemma is an easy adaption from [13], where an analogous statement was proven for the less general case of the QSPP instead of the GSPP.

### Lemma 2

(adapted from [13, Prop. 4]) Let *G* be a $\mathcal {P}_{st}$-covered acyclic digraph with a source vertex *s* and a sink vertex *t*. Let $f: \mathcal {P}_{st}\rightarrow \mathbb {R}$ be a generic cost function and let $N\subseteq A$ be a fixed system of nonbasic arcs. If (*G*, *s*, *t*, *f*) is a linearizable instance of the GSPP, then there exists one and only one linear cost function $c: A \rightarrow \mathbb {R}$ which is both a linearizing cost function and in reduced form.

### Proof

We have to prove both existence and uniqueness. For the existence, by assumption we have that (*G*, *s*, *t*, *f*) is a linearizable instance. Hence there exists a linearizing function $c: A \rightarrow \mathbb {R}$, not necessarily in reduced form. Consider some vertex $v \in V {\setminus } \{{s,t}\}$ and its nonbasic arc $a_v$. Consider the following modification of the function *c*: Let $\beta = c(a_v)$, then reduce the cost of each outgoing arc of *v* by $\beta$, and increase the cost of each incoming arc of *v* by $\beta$. This operation sets the cost of $a_v$ to 0 and does not change the linear cost of any *s*-*t*-path. Now let $v_1,\dots ,v_n$ be a topological vertex order with $v_1 = s$ and $v_n = t$. We repeat the described operation for every vertex $v_{n-1}, v_{n-2}, \dots , v_2$ in this order. It is easily verified that the obtained cost function is a linearization of (*G*, *f*) and is in reduced form.

For the uniqueness, assume that there are two distinct linearizing functions $c, c': A \rightarrow \mathbb {R}$ with the property that all nonbasic arcs have value 0. Consider some topological arc order $\preceq$ and let $a = (u,v)$ be the first arc in the order such that $c(a) \ne c'(a)$. There exists an *s*-*u*-path *P*, because *G* is $\mathcal {P}_{st}$-covered. The path $R:= P \cdot a \cdot N_v$ is an *s*-*t*-path. By assumption, we have $c(P) = c'(P)$ and $c(N_v) = c'(N_v) = 0$. But then $c(R) \ne c'(R)$, a contradiction. $\square$

Let (*G*, *s*, *t*, *f*) be a linearizable instance of the GSPP with $G=(V,A)$ and $N\subseteq A$ be a fixed system of nonbasic arcs. For a linearizing cost function $c: A \rightarrow \mathbb {R}$, we denote by ${{\,\mathrm{\textsf {reduced}}\,}}(c)$ the unique linearizing cost function in reduced form (which exists due to Lemma 2). It follows from the arguments in the proof of Lemma 2 that for given *c* one can compute ${{\,\mathrm{\textsf {reduced}}\,}}(c)$ in $\mathcal {O}(n +m)$ time. We are now ready to prove our main theorem.

### Proof of Theorem 1

The necessity of the conditions for linearizability is trivial. To prove the sufficiency we assume that every two-path system is linearizable with respect to *f* and show that (*G*, *s*, *t*, *f*) is linearizable. Let $N$ be a system of nonbasic arcs. We consider a topological arc order $\preceq$ on the set *A* of arcs in *G* and inductively define a linearizing cost function $c: A \rightarrow \mathbb {R}$ as follows. For any arc $a = (u,v)$ consider some arbitrary *s*-*u*-path *P* and set

4 $$\begin{aligned} c(a) := {\left\{ \begin{array}{ll} f(P \cdot a \cdot N_v) - \sum _{a' \in P}c(a') &{} \text{ if } a \not \in N\\ 0 &{} \text{ otherwise } \end{array}\right. } \end{aligned}$$

The main idea behind this definition is the following: Due to Lemma 2, whenever we look for a linearizing function, we can w.l.o.g. look for one in reduced form. So imagine we have a linearizing function $c'$ such that already $c'(a) = 0$ for all nonbasic arcs. It is not hard to see that Equation (4) is a necessary condition on $c'$ that must be true for every *s*-*u*-path *P* (since all arcs after *a* on the path $P \cdot a \cdot N_v$ have cost 0). This gives us an initial idea to define *c*. Now consider the following claim.

*Claim* If all two-path systems in *G* are linearizable with respect to *f*, then

(i) Function *c* in Equation (4) is well-defined and independent of the concrete choice of *P*.
(ii) The following equation holds for all arcs $(u,v) \in A$ and all *s*-*u*-paths *P*:
  5 $$\begin{aligned} f(P \cdot a \cdot N_v)=c(a)+\sum _{a' \in P} c(a') = c(P \cdot a \cdot N_v) \end{aligned}$$

Observe that the claim immediately implies that (*G*, *s*, *t*, *f*) is linearizable. Indeed, let *c* be the cost function defined in Equation (4) and let *Q* be some *s*-*t*-path. Choose $a = (x,t)$ to be the last arc on *Q*. Then $N_t$ is the trivial path from *t* to *t*, so by applying Equation (5) to the arc *a*, we have $f(Q) = c(Q)$.

**Fig. 2:**
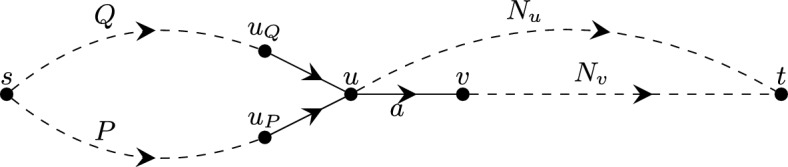
An illustration for the proof of the claim in Theorem 1. The dashed lines represent paths. The arc (*u*, *v*) and the two-path system $(u,P,Q,N_u, a\cdot N_v)$ play a vital role

*Proof of the claim.* We use induction over $\preceq$. For each arc $a = (u, v)$ in *A*, we distinguish between three cases. A sketch of the situation is provided in Fig. 2.

*Case 1* $u = s$. This is the base case of the induction. If *a* is incident to the source vertex, then statement (i) holds, because the only *s*-*u*-path is the trivial path. Statement (ii) holds by the definition of *c*(*a*), and because all nonbasic arcs $a'$ have $c(a') = 0$.

*Case 2* $u \ne s$ *and* $a \not \in N$. Let *a* be basic and not incident to the source. By the induction hypothesis, $c(a')$ is well-defined for all arcs $a'$ preceding *a*. Hence for the proof of statement (i), it remains to show that *c*(*a*) is independent of the choice of *P*. Let *Q* be a second *s*-*u*-path besides *P*, we have to show that

$$\begin{aligned} f(P \cdot a \cdot N_v) - \sum _{a' \in P}c(a') = f(Q \cdot a \cdot N_v) - \sum _{a' \in Q}c(a'). \end{aligned}$$

To see this, let $(u_P, u)$ be the last arc on the path *P*, and let $(u_Q, u)$ be the last arc on the path *Q*, see Fig. 2.

By the induction hypothesis (ii) applied to $(u_P, u)$, we have that $f(P \cdot N_u) = c(P \cdot N_u) = c(P)$, analogously we have $f(Q \cdot N_u) = c(Q \cdot N_u) = c(Q)$. Furthermore, as the two-path system $(u, P, Q, N_u, a \cdot N_v)$ is linearizable, Proposition 1 implies $f(P \cdot N_u) + f(Q \cdot a \cdot N_v) = f(P \cdot a \cdot N_v) + f(Q \cdot N_u)$. Putting everything together, we have

$$\begin{aligned} f(P \cdot a \cdot N_v) - c(P)&= f(Q \cdot a \cdot N_v) + f(P \cdot N_u) - f(Q \cdot N_u) - c(P)\\&= f(Q \cdot a \cdot N_v) + c(P) - c(Q) - c(P)\\&= f(Q \cdot a \cdot N_v) - c(Q), \end{aligned}$$

which proves statement (i). Statement (ii) immediately follows from (i), the definition of *c*(*a*) and the fact that all nonbasic arcs $a'$ have cost $c(a') = 0$.

*Case 3:* $u \ne s$ *and* $a \in N$. Finally, if *e* is nonbasic, then (i) is trivial. Furthermore, let $(u_P, u)$ be the last arc on the path *P* and let $P'$ be the subpath of *P* without the last arc. Because $a \in N$, the two paths $P' \cdot (u_P, u) \cdot N_u$ and $P \cdot a \cdot N_v$ are equal, so (ii) follows by induction applied to the arc $(u_P, u)$. $\square$

Since in general a graph contains exponentially many different two-path systems, Theorem 1 does not seem to lead to an efficient algorithm for the linearization problem LinGSPP at a first glance. However, we show in the next section that this is indeed the case. The arguments are based on a more technical version of Theorem 1 and involve the concept of so-called *strongly basic arcs* and their property $(\pi )$ defined below.

### Definition 4

Let $G = (V, A)$ be an acyclic $\mathcal {P}_{st}$-covered digraph with source vertex *s* and sink vertex *t*. Let $f: \mathcal {P}_{st}\rightarrow \mathbb {R}$ be a generic cost function and let $N \subseteq A$ be a system of nonbasic arcs in *G*. A basic arc (*u*, *v*) is called *strongly basic*, if it is not incident to the source vertex, that is if $u \ne s$.

A strongly basic arc $a = (u,v)$ has the *property* $(\pi )$, if for any *s*-*u*-path *P* the value ${{\,\textrm{val}\,}}(a, P):= f(P \cdot a \cdot N_v) - f(P \cdot N_u)$ does not depend on the choice of *P*.

Thus, if a strongly basic arc $a = (u,v)$ has the property $(\pi )$, we have ${{\,\textrm{val}\,}}(a, P) = {{\,\textrm{val}\,}}(a, Q)$ for any two *s*-*u*-paths *P*, *Q* and this implies the existence of a value ${{\,\textrm{val}\,}}(a):= {{\,\textrm{val}\,}}(a, P)$ for each *s*-*u*-path *P* and ${{\,\textrm{val}\,}}(a)$ is well defined for each strongly basic arc which has the property $(\pi )$.

Finally, note that by definition, the arc set *A* is partitioned into the three disjoint sets of strongly basic arcs, nonbasic arcs, and arcs incident to *s*.

### Lemma 3

Let $G = (V, A)$ be an acyclic $\mathcal {P}_{st}$-covered digraph with source vertex *s* and sink vertex *t*. Let $f: \mathcal {P}_{st}\rightarrow \mathbb {R}$ be a generic cost function and let $N \subseteq A$ be a system of nonbasic arcs in *G*. Then (*G*, *s*, *t*, *f*) is linearizable if and only if every strongly basic arc has the property $(\pi )$. In this case, the mapping $c: A \rightarrow \mathbb {R}$ given by

$$\begin{aligned} c(a) = {\left\{ \begin{array}{ll} {{\,\textrm{val}\,}}(a) &{} a \text { is strongly basic }\\ f(a \cdot N_v) &{} a = (s,v) \text { is incident to }s\\ 0 &{} a \text { is nonbasic} \end{array}\right. } \end{aligned}$$

is a linearizing cost function in reduced form.

### Proof

Let $a = (u, v)$ be a strongly basic arc. We claim that *a* has the property $(\pi )$ iff for any two *s*-*u*-paths *P*, *Q* the two-path system $(u,P,Q,N_u,a \cdot N_v)$ is linearizable with respect to *f*. Indeed, note that by Proposition 1, the two-path system above is linearizable with respect to *f* iff $f(P \cdot a \cdot N_v) + f(Q \cdot N_u) = f(P \cdot N_u) + f(Q \cdot a \cdot N_v)$. The latter equation is equivalent to ${{\,\textrm{val}\,}}(a,Q) = {{\,\textrm{val}\,}}(a,P)$. Recalling that the latter equality holds for every pair of *P*, *Q* iff *a* has the property $(\pi )$ completes the proof of the claim.

Now, assume that some strongly basic arc (*u*, *v*) does not have the property $(\pi )$. Then, there exist two *s*-*u*-paths *P*, *Q* such that ${{\,\textrm{val}\,}}(a,Q) \ne {{\,\textrm{val}\,}}(a,P)$, implying that

the two-path system $(u,P,Q,N_u,a \cdot N_v)$ is not linearizable with respect to *f*. Therefore, (*G*, *s*, *t*, *f*) is not linearizable.

Finally, assume that every strongly basic arc has the property $(\pi )$.

In the proof of Theorem 1 we use the linearizability assumption only for specific two-path systems of the form $(u, P, Q, N_u, a \cdot N_v)$, where $a = (u, v)$ is some strongly basic arc. Thus, if the property $(\pi )$ holds for all strongly basic arcs, then each such specific two-path system is linearizable with respect to *f* and the linearizability of (*G*, *s*, *t*, *f*) follows. Furthermore, the value *c*(*a*) of the linearizing cost function in Equation (4) equals ${{\,\textrm{val}\,}}(a)$ for any arc *a* which is strongly basic, equals $f(a \cdot N_v)$ for any arc (*s*, *v*) incident to *s*, and equals 0 for any nonbasic arc *a*. $\square$

## A linear time algorithm for the LinSPP_d_

In this section, we describe an algorithm which solves the linearization problem for SPP_d_ (LinSPP_d_) in $\mathcal {O}(m^d)$ time, i.e., in linear time with respect ot the input size. The algorithm uses the relationship between the LinSPP_d_ and the *All-Paths-Equal-Cost Problem (APECP)* which we introduce in Sect. 4.1. In Sect. 4.2 we describe the SPP_d_ algorithm and discuss its running time.

### The all paths equal cost problem of order-*d* (APECP_d_)

The All Paths Equal Cost Problem of Order-*d* (APECP_d_) is defined as follows.
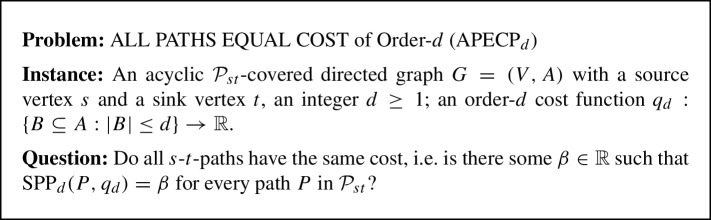


In the following we establish a connection between the LinSPP_d_ and the APECP_d-1_ for $d\ge 2$. More precisely, we show in Lemma 4 that an instance $(G,s,t,q_d)$ of the LinSPP_d_ with an acyclic $\mathcal {P}_{st}$-covered digraph $G=(V,A)$ can be reduced to $\mathcal {O}(m)$ instances of APECP_d-1_, each of them corresponding to exactly one strongly basic arc with respect to some fixed system of nonbasic arcs (see Definitions 3 and 4). The APECP_d-1_ instance corresponding to a strongly basic arc $a = (u, v)$ is defined as follows.

#### Definition 5

Let $d \ge 2$ and let $(G, s,t, q_d)$ be an instance of the LinSPP_d_ with an acyclic $\mathcal {P}_{st}$-covered digraph $G=(V,A)$ and a fixed system of nonbasic arcs *N*. Let $a=(u,v)$ be a strongly basic arc. Denote by $V_u\subseteq V$ the set of vertices lying on at least one *s*-*u*-path and by $A_u\subseteq A$ the set of arcs lying on at least one *s*-*u*-path. Set $G^{(a)}: = (V_u, E_u)$. The instance $I^{(a)}$ of the APECP_d-1_ corresponding to the strongly basic arc *a* is specified by the digraph $G^{(a)}$, the source vertex $s(a):=s$, the sink vertex $t(a):= u$ and the order-$(d-1)$ cost function $q_{d-1}^{(a)}: \{ B \subseteq A_u: |B| \le d-1\} \rightarrow \mathbb {R}$ defined by means of the function $q_d$ as follows:

6 $$\begin{aligned} q_{d-1}^{(a)}(B) := \left( \sum _{\begin{array}{c} C \subseteq N_u\\ |C| \le d - |B| \end{array}}q_d(B \cup C) \right) -\left( \sum _{\begin{array}{c} C \subseteq a \cdot N_v\\ |C| \le d - |B| \end{array}}q_d(B \cup C) \right) . \end{aligned}$$

#### Lemma 4

Let $d \ge 2$ and let $(G, s,t, q_d)$ be an instance of the LinSPP_d_ with an acyclic $\mathcal {P}_{st}$-covered digraph $G=(V,A)$ and a fixed system of nonbasic arcs *N*. The APECP_d-1_ instance $I^{(a)}$ corresponding to some strongly basic arc *a* is a YES-instance iff the arc *a* has the property $(\pi )$ with respect to $f: \mathcal {P}_{st}\rightarrow \mathbb {R}$ given by $f(P)=SPP_d(P,q_d)$ for $P\in \mathcal {P}_{st}$. In this case, ${{\,\textrm{val}\,}}(a) =\beta _a$, where $\beta _a$ is the common cost of all source-sink-paths in the APECP_d-1_ instance $I^{(a)}$.

#### Proof

Let $a = (u,v) \in A$ be a strongly basic arc and let *P* be some *s*-*u*-path in *G*. Then *P* is contained in the graph $G^{(a)} = (V_u, A_u)$. Denote $f^{(a)}(P):=SPP_{d-1}(P,q^{(a)}_{d-1})$ for any *s*-*u*-path *P* in *G*. We have that

$$\begin{aligned}&{{\,\textrm{val}\,}}(a, P) = f_q(P \cdot N_u) - f_q(P \cdot a \cdot N_v) \\&\quad = \sum _{\begin{array}{c} F \subseteq P \cdot N_u\\ |F| \le d \end{array}}q_d(F) - \sum _{\begin{array}{c} F \subseteq P \cdot a \cdot N_v\\ |F| \le d \end{array}}q_d(F)\\&\quad = \sum _{k=0}^d \sum _{\begin{array}{c} B \subseteq P\\ |B| = k \end{array}} \sum _{\begin{array}{c} C \subseteq N_u\\ |C| \le d - k \end{array}}q_d(B \cup C) - \sum _{k=0}^d \sum _{\begin{array}{c} B \subseteq P\\ |B| = k \end{array}} \sum _{\begin{array}{c} C \subseteq a \cdot N_v\\ |C| \le d - k \end{array}}q_d(B \cup C)\\&\quad = \sum _{k=0}^{d-1} \sum _{\begin{array}{c} B \subseteq P\\ |B| = k \end{array}} \sum _{\begin{array}{c} C \subseteq N_u\\ |C| \le d - k \end{array}}q_d(B \cup C)\\&\qquad - \sum _{k=0}^{d-1} \sum _{\begin{array}{c} B \subseteq P\\ |B| = k \end{array}} \sum _{\begin{array}{c} C \subseteq a \cdot N_v\\ |C| \le d - k \end{array}}q_d(B \cup C) \quad + (1 - 1)\sum _{\begin{array}{c} B \subseteq P\\ |B| = d \end{array}}q_d(B)\\&\quad = \sum _{k=0}^{d-1} \sum _{\begin{array}{c} B \subseteq P\\ |B| = k \end{array}} \left( \sum _{\begin{array}{c} C \subseteq N_u\\ |C| \le d - k \end{array}}q_d(B \cup C) - \sum _{\begin{array}{c} C \subseteq a \cdot N_v\\ |C| \le d - k \end{array}} q_d(B \cup C)\right) \\&\quad = \sum _{\begin{array}{c} B \subseteq P\\ |B| \le d-1 \end{array}} q^{(a)}_{d-1}(B)= f^{(a)}(P). \end{aligned}$$

We conclude that the value ${{\,\textrm{val}\,}}(a, P)$ is independent of *P*, if and only if for every path the quantity $f^{(a)}(P)$ does not depend on *P*. The latter condition is equivalent to $I^{(a)}$ being a YES-instance of the APECP_d-1_. Furthermore, if this is the case, then ${{\,\textrm{val}\,}}(a) =\beta _a= f^{(a)}(P)$ for any *s*-*u*-path *P*. $\square$

Lemmas 3 and 4 imply that an instance $(G,s,t,q_d)$ of the SPP_d_ with an acyclic digraph *G* is linearizable iff each instance $I^{(a)}$ of the APECP_d-1_ corresponding to some strongly basic arc *a* (with respect to some fixed system of nonbasic arcs) is a YES-instance. Furthermore, the same lemmas imply that in this case a linearizing cost function in reduced form is obtained by letting $c(a)=\beta _a$ for all strongly basic arcs, $c(a) = 0$ for all nonbasic arcs, and $c(a) = \text {SPP}_d(a \cdot N_v)$ for all arcs $a=(s,v)$ incident to the source.

Thus, we have shown that an instance of the LinSPP_d_ can be reduced to $\mathcal {O}(m)$ instances of the APECP_d-1_. Next, in Lemma 5 we show that each instance of the APECP_d-1_ can be reduced to an instance of the LinSPP_d-1_. First, we define a specific cost function as follows. Let $G = (V, A)$ be a $\mathcal {P}_{st}$-covered acyclic digraph and $\beta \in \mathbb {R}$. The function ${\textsf {source}}_\beta : A \rightarrow \mathbb {R}$ assigns cost $\beta$ to every arc incident to the source *s*, and 0 to all other arcs.

#### Lemma 5

Let $G = (V, A)$ be a $\mathcal {P}_{st}$-covered acyclic digraph with source vertex *s* and sink vertex *t* and let $N \subseteq A$ a fixed system of nonbasic arcs. Let $q_d$ be an order-*d* cost function. The instance $(G,s,t,q_d)$ of the APECP_d_ problem is a YES-instance iff the instance $(G,s,t, q_d)$ of SPP_d_ is linearizable and ${\textsf {source}}_\beta$ is its unique linearizing function in reduced form (with respect to *N*).

#### Proof

Clearly, ${\textsf {source}}_\beta$ is a linearizing function iff all paths have the same cost $\beta$. Furthermore, observe that all arcs incident to the source do not belong to *N*. Therefore ${\textsf {source}}_\beta$ is in reduced form with respect to *N*. In fact, by Lemma 2${\textsf {source}}_\beta$ is the unique linearizing functions in reduced form, and ${{\,\mathrm{\textsf {reduced}}\,}}(c') ={\textsf {source}}_\beta$ for all other linearizing functions $c'$. $\square$

Finally, we show in Lemma 6

#### Lemma 6

The APECP_1_ can be solved in linear time $\mathcal {O}(m)$.

#### Proof

This is an easy exercise in dynamic programming. The algorithm uses the fact that in a $\mathcal {P}_{st}$-covered acyclic digraph all *s*-*t*-paths have the same cost if and only for every vertex *w* all *s*-*w*-paths have the same cost. Hence we can introduce a variable $y_w \in \mathbb {R}$ for every vertex *w*. We let $y_s = 0$ and then check in topological vertex order for every vertex *w*, whether the value $y_u + q_1(\{{(u,w)}\})$ is the same for every incoming edge (*u*, *w*). Finally, if this is the case, the common cost of all source-sink paths is given by $y_t + q_1(\emptyset )$. $\square$

We remark that attention to the case $q_1(\emptyset ) \ne 0$ is crucial. This is because by definition, the term $q_1(\emptyset )$ is always included in SPP$_1(P, q_1)$ for every possible path *P*. Indeed, Equation (6) may produce instances with the property $q_1(\emptyset ) \ne 0$.

### The linear time LinSPP_d_ algorithm

Our LinSPP_d_ algorithm ${\mathcal {A}}$ works as follows. Consider an instance $(G,s,t,q_d)$ of the LinSPP_d_ with an acyclic $\mathcal {P}_{st}$-covered digraph *G*, with source vertex *s*, sink vertex *t* and order-*d* cost function $q_d$. We first fix some system of nonbasic arcs *N* and construct the instance $I^{(a)}$ of the APECP_d-1_ problem given in Definition 5 for each strongly basic arc *a*. Then, we check each instance $I^{(a)}$ for being a YES-instance and do this by reducing $I^{(a)}$ to an instance of LinSPP_d-1_ according to Lemma 5. By iterating this process we eventually end up with instances of APECP_1_ which can be easily solved by dynamic programming, as demonstrated in Lemma 6. A summary in pseudocode is provided in Algorithm 1. The correctness of the algorithm follows from Lemmas 3 to 6.

**Algorithm 1:**
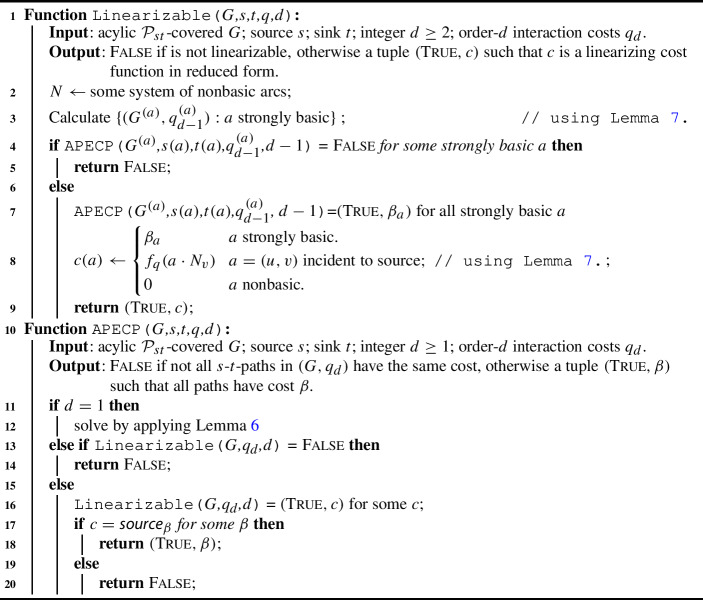
An algorithm to solve the LinSPP_d_.

It is not hard to implement the algorithm described above in $\mathcal {O}(d^2m^{d+1})$ time. With a careful implementation it is possible to achieve a better result as stated in Theorem 2.

#### Theorem 2

The LinSPP_d_ on acyclic digraphs can be solved in $\mathcal {O}(d^2m^d)$ time.

Note that the input size required to encode the cost function $q_d$ equals . Thus, $\mathcal {O}(d^2m^d)$ is linear in the input size and hence optimal if *d* is considered a constant, like for example in the QSPP. The remainder of the section is devoted to the proof of Theorem 2. The main bottleneck we have to get rid of is the computation of the instances $I^{(a)}$ corresponding to the strongly basic arcs *a* in line 3 of Algorithm 1. If one simply uses the definition of $q^{(a)}_{d-1}$ from Equation (6), then one can see that for each *a* one can compute $q^{(a)}_{d-1}$ in $\mathcal {O}(dm^d)$ time. As this needs to be repeated for each strongly basic arc *a*, in total this would take $\mathcal {O}(dm^{d+1})$ time. A similar bottleneck arises in line 8 when computing $f_q(a \cdot N_v)$. We get rid of these two bottlenecks by calculating some *auxiliary values*.

For all sets $B \subseteq A$ of arcs with $|B| \le d-1$ and all vertices $x \in V \setminus \{{s}\}$, we define the value

7 $$\begin{aligned} \gamma (B, x) := \sum _{\begin{array}{c} C \subseteq N_x\\ |C| \le d - |B| \end{array}}q_d(B \cup C). \end{aligned}$$

#### Lemma 7

The line 3 and the line 8 of Algorithm 1 can be implemented such that their execution takes at most $c d m^d$ steps for some constant $c \ge 0$, independent of both *m* and *d*.

#### Proof

Consider the auxiliary values $\gamma (B,x)$ from Equation (7). We show how to compute all the values of $\gamma$ for $B \subseteq A, |B| \le d-1, x \in V {\setminus } \{{s}\}$ in $\mathcal {O}(dm^d)$ time. We begin by showing how to compute the value $\gamma (B, x)$ for all vertices $x \in V\setminus \{{s}\}$ and for some fixed set $B \subseteq A$ with $|B| \le d-1$. Let $k = |B|$ be the size of *B*, $k \in \{{{0}, \ldots , {d-1}}\}$. Assume that $\gamma (B, v)$ has already been computed for some nonbasic arc $a = (u, v) \in N$. Then, $\gamma (B,u)$ can be computed as follows. Since $a \in N$, we have $N_u = a \cdot N_v$. Moreover, the following equalities hold

8 $$\begin{aligned} \gamma (B, u)&= \sum _{\begin{array}{c} C \subseteq a \cdot N_v\\ |C| \le d - |B| \end{array}}q_d(B \cup C) \nonumber \\&= \sum _{\begin{array}{c} C \subseteq N_v\\ |C| \le d - |B| - 1 \end{array}} q_d(B \cup \{{a}\} \cup C) + \sum _{\begin{array}{c} C \subseteq N_v\\ |C| \le d - |B| \end{array}}q_d(B \cup C)\nonumber \\&= \gamma (B, v) + \sum _{\begin{array}{c} C \subseteq N_v\\ |C| \le d - |B| - 1 \end{array}}q_d(B \cup \{{a}\} \cup C). \end{aligned}$$

This formula can be evaluated in $$\left( {\begin{array}{c}m\\ {d - k - 1}\end{array}}\right) $$ time. We can now traverse the tree of nonbasic arcs, starting at the root *t*, where $\gamma (B, t) = q(B)$, and iteratively apply the formula (8) to compute all values $\gamma (B, x)$ for the vertices $x \in V \setminus \{{s}\}$. Thus, it takes $$n\left( {\begin{array}{c}m\\ {d - k - 1}\end{array}}\right) $$ time to compute all values $\gamma (B, x)$ for a fixed set *B* of size *k*. For each $k = 0,\dots ,d-1$, there are $$\left( {\begin{array}{c}m\\ k\end{array}}\right) $$ subsets of size *k*. Therefore, the total time to compute all values of $\gamma$ is

$$\begin{aligned} \sum _{k=0}^{d-1} n\left( {\begin{array}{c}m\\ d - k - 1\end{array}}\right) \left( {\begin{array}{c}m\\ k\end{array}}\right) \le \sum _{k=0}^d n\frac{m^{d-k-1}}{(d-k-1)!} \frac{m^k}{k!} \le (d+1) nm^{d-1}. \end{aligned}$$

(Note that here we used the very rough estimation $t! \ge 1$.) As the digraph is $\mathcal {P}_{st}$-covered, we can assume w.l.o.g. that it is connected, implying $n\le 2m$ and consequently, ($d+1) nm^{d-1}\le 4dm^d$. Thus, there exists a constant $c_1 \ge 0$, independent on *n* and *d*, such that all needed values of $\gamma$ can be computed in $c_1dm^d$ time.

Now assume that all the values $\gamma (B, x)$ have been computed. For every strongly basic arc $a = (u, v)$, and each set $B \subseteq A_u$ with $|B| \le d-1$, the order-$d-1$ cost function in the corresponding APECP_d-1_ instance fulfills

$$\begin{aligned} q_{d-1}^{(a)}(B) = \gamma (B,u) - \gamma (B, v) - \sum _{\begin{array}{c} C \subseteq N_v\\ |C| \le d - |B| - 1 \end{array}}q_d (B \cup \{{a}\} \cup C). \end{aligned}$$

This can be seen by plugging the definition of $\gamma$ into Equation (6). This equation can be evaluated in $$\left( {\begin{array}{c}m\\ d - k -1\end{array}}\right) $$ time if $k=0,\dots ,d-1$ is the size of *B*. In order to obtain all the desired values for $q^{(a)}_{d-1}(B)$ for all the APECP_d-1_ instances, we need to consider every choice of the set *B* (again there are at most $$\left( {\begin{array}{c}m\\ k\end{array}}\right) $$ sets of size *k*) and every of the at most $\mathcal {O}(m)$ choices of the arc *a*. The same analysis as before shows that given the computed values of $\gamma$, all required values of $q^{(a)}_{d-1}(B)$ can be computed in at most $c_3dm^d$ steps for some constant $c_3 \ge 0$. Finally, computing the vertex set $V_u$ and arc set $A_u$ of the APECP_d-1_ instance clearly can be done in linear time $\mathcal {O}(m)$ for each strongly basic arc $a = (u,v)$. We conclude that computing the set $\{{(G^{(a)}, q^{(a)}_{d-1}): a \text { strongly basic })}\}$ of digraphs for all APECP_d-1_ instances can be done in at most $c_4dm^d$ steps for some constant $c_4 \ge 0$.

Finally consider line 8 of Algorithm 1 and assume that all auxiliary values $\gamma (B,x)$ have been computed. Let $a = (s,v)$ be an arc incident to the source. We have that

$$\begin{aligned} f_q(a \cdot N_v) = \gamma (\emptyset , v) +\sum _{\begin{array}{c} C \subseteq N_v\\ |C| \le d - 1 \end{array}}q(\{{a}\} \cup C). \end{aligned}$$

This formula can be evaluated in $\mathcal {O}(m^{d-1})$ time for each arc *a*. We conclude that the set of all values $f_q(a \cdot N_v)$ in line 8 of Algorithm 1 can be computed in at most $c_5m^d$ steps for some constant $c_5 \ge 0$, thus completing the proof of the lemma. $\square$

#### Proof of Theorem 2

It follows from Lemmas 3 to 6 that Algorithm 1 correctly solves the LinSPP_d_. It follows from Lemma 7 that lines 3 and 8 can be implemented to take at most $c_0dm^d$ steps each for some constant $c_0 \ge 0$. The running time of all the remaining lines is asymptotically dominated by $m^d$. Now let *f*(*m*, *d*) denote the worst-case running time of $\texttt {Linearizable()}$ on an instance with *m* arcs and an order-*d* cost function. Let *g*(*m*, *d*) be the corresponding function for $\texttt {APECP()}$. By the preceding arguments, there exists a constant $c \ge 0$ such that the following inequalities hold:

$$\begin{aligned} f(m ,d)&\le cdm^d + mg(m, d-1);&d \ge 2\\ g(m, d)&\le cm + f(m, d);&d \ge 2\\ g(m, 1)&\le cm. \end{aligned}$$

By induction over *d* it is easy to see that $f(m, d) \le cd(2d - 2)m^d$ and $g(m, d) \le cd(2d - 1)m^d$. Indeed, in the base case $d = 1$ we have $g(m, 1) \le cm$. For $d \ge 2$ we have

$$\begin{aligned} f(m, d)&\le cdm^d + mc(d-1)(2d - 3)m^{d-1} \le cd(2d - 2)m^d \\ g(m, d)&\le cm + cd(2d - 2)m^d \le cd(2d -1)m^d. \end{aligned}$$

This implies that $f = \mathcal {O}(d^2m^d)$, which was to show. $\square$

## The subspace of linearizable instances

Let $d\in \mathbb {N}$, $d\ge 2$, and an acyclic, $\mathcal {P}_{st}$-covered digraph $G = (V, A)$ with source vertex *s* and sink vertex *t* be fixed. Let $H^{(d)}:= \{{B \subseteq H \mid |B| \le d}\}$ be the set of all subsets of at most *d* arcs in arc set $H\subseteq A$. Every order-*d* cost function $q_d: A^{(d)}\rightarrow \mathbb {R}$ can be uniquely represented by a vector $x \in \mathbb {R}^{A^{(d)}}$ with $q_d(F)=x_F$ for all $F\in A^{(d)}$, and vice-versa. Thus, each instance $(G,s,t,q_d)$ can be identified with the corresponding vector $x\in \mathbb {R}^{A^{(d)}}$ and we will say that $x\in \mathbb {R}^{A^{(d)}}$ is an instance of the SPP_d_. In this context, an instance $x\in \mathbb {R}^{A^{(d)}}$ of the SPP_d_ is linearizable iff there exists an $\bar{x} =(x_a)_{a\in A} \in \mathbb {R}^{A^{(1)}}$ such that $\sum _{S\subseteq P: |S|\le d} x_S=\sum _{a\in P}\bar{x}_a$ for all *s*-*t*-paths *P*. (Note that sometimes we will slightly abuse the notation and use *A* instead of $A^{(1)}$.) Thus, clearly, if $x,y \in \mathbb {R}^{A^{(d)}}$ are linearizable instances of the SPP_d_, then $\mu x + \nu y$ is also a linearizable instance, for all scalars $\mu ,\nu \in \mathbb {R}$. Therefore, the set of linearizable instances of the SPP_d_ on the fixed digraph *G* forms a linear subspace ${\mathcal {L}}_d$ of $\mathbb {R}^{A^{(d)}}$.

Methods to compute this subspace are useful in B &B algorithms for the SPP_d_ as they can be applied to compute better lower bounds along the lines of what Hu and Sotirov [13] did for general quadratic binary programs. Hu and Sotirov showed that for $d = 2$ a basis of ${\mathcal {L}}_d$ can be computed in polynomial time [13, Prop. 5]. We extend their result to the case $d > 2$.

### Theorem 3

Let $G = (V, A)$ be an acyclic, $\mathcal {P}_{st}$-covered digraph with source vertex *s* and sink vertex *t* and let $d \in \mathbb {N}$ be a constant. A basis of the subspace ${\mathcal {L}}_d$ of the linearizable instances of the SPP_d_ on *G* can be computed in polynomial time.

The goal of this section is to prove Theorem 3. The main idea of the proof is that each line, and subsequently each block of Algorithm 1 can be interpreted as a linear function *f*, that maps the input variables linearly to some output vector, such that *f* outputs the 0-vector iff the input to *f* is a YES-instance. Hence if we are able to show how to compute *f* for all basis vectors $e_1,\dots e_k$, then we can compute a matrix *M* describing the function *f*. By computing the kernel of this matrix, we obtain the desired linear subspace.

More formally, in our proof we specify an integer $k\in \mathbb {N}$ bounded by a polynomial in the size of the input of the SSP_d_ on $G=(V,A)$ and a matrix $M\in \mathbb {R}^{k\times |A^{(d)}|}$ such that for $f: \mathbb {R}^{A^{(d)}} \rightarrow \mathbb {R}^k$ with $f(x)=Mx$, we have: $f(x) = 0$ iff *x* is a linearizable instance of the SPP_d_ on *G*. Thus, the linearizable instances *x* of the SPP_d_ on *G* form the kernel of *M*, which can be efficiently computed. If there are no contextual ambiguities we briefly say that the integer *k* as above is *polynomially bounded*.

The function *f* will be composed of smaller building blocks, mimicking the way that our algorithm from Sect. 4.2 reduces the SPP_d_ to smaller instances of the APECP_d-1_, which in turn are reduced to instances of the SPP_d-1_. There are four different kinds of building blocks, which we denote by $$f_{u,d}, f'_{u,d}, g_{u,d},$$ and $$ g'_{u,d}$$. Formally, for a vertex *u* let $$V_u$$ ($$A_u$$) be the set of vertices (arcs) lying on at least one *s*-*u*-path, as specified in Definition 5. An instance of the SPP_d_ on the smaller digraph $$(V_u, A_u)$$ with source *s* and sink *u* can be interpreted as a vector $$x \in \mathbb {R}^{A_u^{(d)}}$$. Likewise, an instance of the APECP_d_ on the smaller digraph $$(V_u, A_u)$$ can be interpreted as a vector $$x \in \mathbb {R}^{A_u^{(d)}}$$. In the following, we show that for all vertices $$u \in V$$ and every $$d \in \mathbb {N}, d\ge 2$$, there exists some polynomially bounded $$k \in \mathbb {N}$$ and a linear function

$$\begin{aligned} f_{u,d}: \mathbb {R}^{A_u^{(d)}} \rightarrow \mathbb {R}^k \end{aligned}$$

such that $f_{u,d}(x) = 0$ if and only if *x* is a YES-instance of the linSPP_d_ on the digraph $(V_u, A_u)$. Furthermore, if an instance $x \in \mathbb {R}^{A_u^{(d)}}$ is linearizable, we would like to use its linearizing function as a new building block. Hence we prove the existence of a linear function $f'_{u,d}$, such that whenever $f_{u,d}(x) = 0$ for some instance *x*, then $f'_{u,d}(x) \in \mathbb {R}^{A_u}$ is a linearizing function of the instance *x*.

Likewise, we show that for all vertices $u \in V$ and every $d \in \mathbb {N}, d\ge 1$, there exists some polynomially bounded $k \in \mathbb {N}$ and a linear function

$$\begin{aligned} g_{u,d}: \mathbb {R}^{A_u^{(d)}} \rightarrow \mathbb {R}^k \end{aligned}$$

such that $g_{u,d}(x) = 0$ if and only if *x* is a YES-instance of the APECP_d_ on the digraph $(V_u, A_u)$. Similar to above, we prove the existence of a linear function $g'_{u,d}$, such that whenever $g_{u,d}(x) = 0$ for some instance *x*, then $g'_{u,d}(x) \in \mathbb {R}$ is the common cost of all paths in the instance *x*.

We prove the two claims stated above by induction. The base case of the induction is concerned with the APECP_1_. For the remainder of this section, we consider a fixed acyclic and $\mathcal {P}_{st}$-covered digraph $G = (V, A)$ with source vertex *s* and sink vertex *t* with *n* vertices and *m* arcs.

### Lemma 8

Consider the APECP_1_ on the digraph $(V_u, A_u)$ with source *s* and sink *u* for some vertex $u\in V$. The following statements hold:

(i) There exists $k \in \mathbb {N}$ and a linear function $g_{u,1}: \mathbb {R}^{A_u^{(1)}} \rightarrow \mathbb {R}^k$, such that $k = \mathcal {O}(m)$ and such that $g_{u,1}(x) = 0$ iff *x* is a YES-instance of the APECP_1_.
(ii) There exists a linear function $g'_{u,1}: \mathbb {R}^{A_u^{(1)}} \rightarrow \mathbb {R}$, such that for all *x* with $g_{u,1}(x) = 0$, we have that $g'_{u,1}(x)$ is the common cost of all *s*-*u*-paths in the APECP_1_ instance *x*.
(iii) The functions $g_{u,1}, g'_{u,1}$ can be evaluated in $\mathcal {O}(m)$ time, given an input $x \in \mathbb {R}^{A_u^{(1)}}$.

### Proof

Assume we are given an instance $x \in \mathbb {R}^{A_u^{(1)}}$. The vector *x* represents a function $x: A_u^{(1)} \rightarrow \mathbb {R}$. Sometimes we use the equivalent notation *x*(*F*) instead of $x_F$, for $F\in A_u^{(1)}$. We consider the same dynamic program as in Lemma 6. For every vertex $w \in V_u$, we fix some *s*-*w*-path $P_w$. Furthermore, we make sure that the set of all paths $P_w$ chosen forms an out-tree rooted at *s*, that is, $\bigcup _{w \in V_u} P_w$ forms a tree. We introduce an auxiliary variable $y_w$ for every vertex $w \in V_u$ by making use of $P_w$:

9 $$\begin{aligned} y_w := \sum _{a \in P_w}x(\{{a}\})\, . \end{aligned}$$

Clearly, $y_w$ depends linearly on *x*. By the same argumentation as in Lemma 6, the dynamic program correctly concludes that the APECP_1_ instance is a YES-instance iff

$$\begin{aligned} \forall a = (w,z) \in A_u: y_z = y_w + x(\{{a}\}). \end{aligned}$$

Further, if *x* is a YES-instance, then the common cost of all *s*-*u*-paths equals $y_u + x(\emptyset )$. (Note that the case $x(\emptyset ) \ne 0$ can appear.)

Now we construct the functions $g_{u,1}, g'_{u,1}$ in the following way. $g_{u,1}$ maps to $\mathbb {R}^k$ with $k = |A_u|$ and is given by

$$\begin{aligned} g_{u,1}(x) = ( y_w + x(\{{a}\}) - y_z)_{a = (w,z) \in A_u}\,, \text{ for } \text{ all }\ x\in \mathbb {R}^{A_u^{(1)}}. \end{aligned}$$

The real-valued function $g'_{u,1}$ is given by $g'_{u,1}(x) = y_u + x(\emptyset )$, for all $x\in \mathbb {R}^{A_u^{(1)}}$.

Since $y_w$ depends linearly on *x*, for any $w\in V_u$, both functions $g_{u,1}, g'_{u,1}$ are linear. The properties stated in (i) and (ii) are fulfilled by construction. Further, it is straightforward to see that given an input $x \in \mathbb {R}^{A_u^{(1)}}$, the value $g'_{u,1}(x)$ can be computed in $\mathcal {O}(m)$ time. Finally, note that the vector $g_{u,1}(x)$ can be computed in $\mathcal {O}(m)$ time. In order to achieve this, we can make use of the simple recursive formula $y_w = y_z + x(\{{(z,w)}\})$, where (*z*, *w*) is the last arc on path $P_w$. This formula is correct, because the paths $P_w$ form a tree. $\square$

### Lemma 9

Let $d \ge 2$. Consider the SPP_d_ and the APECP_d_ on the digraph $(V_u, A_u)$ with source *s* and sink *u* for some vertex $u\in V$. The following statements hold:

(i) There exists $k \in \mathbb {N}$, $k = \mathcal {O}(m^{d})$, and a linear function $f_{u,d}: \mathbb {R}^{A_u^{(d)}} \rightarrow \mathbb {R}^k$, such that $f_{u,d}(x) = 0$ iff *x* is a linearizable instance of the SPP_d_.
(ii) There exists a linear function $f'_{u,d}: \mathbb {R}^{A_u^{(d)}} \rightarrow \mathbb {R}^{A_u}$, such that for all *x* with $f_{u,d}(x) = 0$, $f'_{u,d}(x) \in \mathbb {R}^{A_u}$ is a linearizing cost function in reduced form for the SPP_d_ instance *x*.
(iii) There exists $k \in \mathbb {N}$, $k = \mathcal {O}(m^{d})$, and a linear function $g_{u,d}: \mathbb {R}^{A_u^{(d)}} \rightarrow \mathbb {R}^k$, such that $g_{u,d}(x) = 0$ iff *x* is a YES-instance of the APECP_d_.
(iv) There exists a linear function $g'_{u,d}: \mathbb {R}^{A_u^{(d)}} \rightarrow \mathbb {R}$, such that for all *x* with $g_{u,d}(x) = 0$, $g'_{u,d}(x)$ is the common cost of all *s*-*u*-paths in the APECP_d_ instance *x*.
(v) The functions $f_{u,d}, f'_{u,d}, g_{u,d}, g'_{u,d}$ can be evaluated in $\mathcal {O}(m^d)$ time, given an input $x \in \mathbb {R}^{A_u^{(d)}}$.

### Proof

We prove the lemma by induction. Lemma 8 shows that the statements in (iii) and (iv) hold for $d = 1$. For the inductive step with $d\ge 2$ assume that the statements in (iii) and (iv) hold for the value $d - 1$. Thus, the functions $g_{u,d-1}$ and $g'_{u,d-1}$ fulfilling the properties in (iii) and (iv) exist. We now proceed to show that the statements in (i) – (iv) hold for the value *d*, in particular, we construct the functions $f_{u,d}, f'_{u,d}, g_{u,d}, g'_{u,d}$ with the corresponding properties.

Consider the order-*d* cost function $q_d \in \mathbb {R}^{A_u^{(d)}}$ of an SPP_d_ instance (APECP_d_ instance) on the graph $(V_u, A_u)$ with source *s* and sink *u*. For a fixed vertex *u* such instances of the SPP_d_ (APECP_d_) are fully determined by $q_d$. Hence, unless other specified, we will identify SPP_d_ instances (APECP_d_ instances) as above with the corresponding order-*d* cost function $q_d$. Fix some system $N \subseteq A_u$ of nonbasic arcs with respect to $(V_u, A_u)$. Let $F \subseteq A_u$ be the set of strongly basic arcs with respect to *N*, and let $F'$ be the set of arcs which are incident to the source (hence $A_u = N \dot{\cup }F \dot{\cup }F'$ is a partition by the definition of basic/strongly basic arcs). Lemmas 3 and 4 imply that $q_d$ is a YES-instance of LinSPP_d_ iff $q^{(a)}_{d-1}$ is a YES-instance of APECP_d-1_, for every arc $a = (w,z) \in F$. By the inductive assumption, the latter is the case iff $g_{w,d-1}(q^{(a)}_{d-1}) = 0$. Observe that the function which maps $q_d \in \mathbb {R}^{A_u^{(d)}}$ to $q^{(a)}_{d-1} \in \mathbb {R}^{A_w^{(d-1)}}$, $a\in F$, is a linear function (this follows from the definition, i.e. Equation (6)). Therefore, the composition $f_{u,d}$ defined by

$$\begin{aligned} f_{u,d}(q_d) = (g_{w,d-1}(q^{(a)}_{d-1}))_{a = (w,z) \in F}, \end{aligned}$$

is linear.

The inductive assumption implies that $g_{w,d-1}(q^{(a)}_{d-1})\in \mathbb {R}^{k'}$ with $k'=\mathcal {O}(m^{d-1})$, for each $a=(w,z)\in F$. Since $|F|=\mathcal {O}(m)$, the composition $f_{u,d}$ maps to $\mathbb {R}^k$ with $k = \mathcal {O}(m k') = \mathcal {O}(m^d)$. Since the instance $q_d$ of the SPP_d_ is linearizable iff $g_{w,d-1}(q^{(a)}_{d-1}) = 0$, for all $a\in F$, property (i) is satisfied. Further, we define $f'_{u,d}: \mathbb {R}^{A_u^{(d)}}\rightarrow \mathbb {R}^{A_u}$ by setting $f'_{u,d}(q_d)=(x_a)_{a\in A_u}\in \mathbb {R}^{A_u}$ with

$$\begin{aligned} x_a = {\left\{ \begin{array}{ll} g'_{w,d-1}(q^{(a)}_{d-1}) &{} a = (w,z) \in F\\ 0 &{} a \in N\\ \text {SPP}_d(a \cdot N_z, q_d) &{} a = (w,z) \in F'\, . \end{array}\right. } \\ \end{aligned}$$

Then $f'_{u,d}(q_d)$ is a mapping of $A_u$ to $\mathbb {R}$ and it is a linearizing cost function for the $q_d$ instance of the SPP_d_ whenever $f_{u,d}(q_d) = 0$. Moreover, this linearizing function is in reduced form. These facts follows from the arguments in Lemmas 3 and 4 (see also the description right after the proof of Lemma 4) and the inductive assumption on $g'_{w,d-1}$. Further, observe that the function $f'_{u,d}$ is linear by construction, since $q_{d-1}^{(a)}$ and $g'_{w,d-1}$ are linear for all $a=(w,z)\in F$. By applying the speedup technique from Sect. 4.2 the sequence of values $(q^{(a)}_{d-1})_{a \in F}$, as well as the sequence $(\text {SPP}_d(a \cdot N_z; q_d))_{a\in F}$ can be computed in $\mathcal {O}(m^d)$ time for any given $q_d$. Combined with the inductive assumption that both $g_{w,d-1}, g'_{w,d-1}$ can be computed in $\mathcal {O}(m^{d-1})$ time for each vertex *w*, we obtain that both $f_{u,d}, f'_{u,d}$ can be computed in $\mathcal {O}(m^d)$ time. Hence we have shown (i) and (ii).

Finally, we show how to construct $g_{u,d}, g'_{u,d}$ while assuming that the the functions $f_{u,d}$, $f'_{u,d}$ described in (i) and (ii) exists and have the corresponding properties. Let $q_d \in \mathbb {R}^{A_u^{(d)}}$ be an instance of the APECP_d_. It follows by the arguments from Lemma 5 that $q_d$ is a YES-instance with all *s*-*u*-paths having cost $\beta$ iff $q_d$ is a linearizable instance of SPP_d_. Moreover, in the linearizable case, the equality ${{\,\mathrm{\textsf {reduced}}\,}}(c) = {\textsf {source}}_\beta$ holds for the linearizing function *c*. Then, the inductive assumption and the uniqueness of the reduced form (see Lemma 2) imply that $q_d$ is a YES-instance of the APECP_d_ iff the following equalities hold

10 $$\begin{aligned}&\qquad f_{u,d}(q_d) = 0 \nonumber \\&\text { and } \forall a,a' \in F': (f'_{u,d}(q_d))(a) = (f'_{u,d}(q_d))(a') \nonumber \\&\text { and } \forall a \in A_u \setminus F': (f'_{u,d}(q_d))(a) = 0. \end{aligned}$$

Then, we construct $g_{u,d}: \mathbb {R}^{A_u^{(d)}}\rightarrow \mathbb {R}^{k'}$ with $k'=k + p+q$, $p:=|F'|(|F'|-1)/2$ and $q:=|A_u^{(d)}|-|F'|$ as follows. We denote $g_{u,d}(q_d)=(x_i)_{i=1,2,\ldots ,k'}$ and specify the first *k* entries of *x* by $(x_i)_{i=1,2,\ldots ,k}:=f_{u,d}(q_d)$. If $p\ne 0$, the next *p* entries of *x* are specified by means of an arbitrary but fixed ordering of the two-element subsets $\{a,a'\}\subseteq F'$: $x_{k+i}=(f'_{u,d}(q_d))(a_i) -(f'_{u,d}(q_d))(a'_i)$, $i=1,2,\ldots ,p$, where $\{a_i,a'_i\}$ is the i-th subset with respect to the above ordering. Finally, if $q\ne 0$, the entries $x_i$, $i=k+p+1,\ldots , k'$ are specified by means of an arbitrary but fixed ordering of the arcs in $A_u {\setminus } F'$: set $x_{k+p+i}=(f'_{u,d}(q_d))(a_i)$, $i=1,2,\ldots ,q$, where $a_i$ is the i-th arc with respect to the above ordering. Trivially, $g_{u,d} = 0$ iff all three of the above conditions in Equation (10) are satisfied, thus iff $q_d$ is a YES-instance of the APECP_d_. Moreover, we have $k'=\mathcal {O}(m^d)$, since $p=\mathcal {O}(m^d)$, $p=\mathcal {O}(m^d)$ and $k = \mathcal {O}(m^d)$ due to the inductive assumption on $f_{u,d}$. Thus $g_{u,d}$ fulfills the properties stated in (iii). Further, we define $g'_{u,d}(q_d):=(f'_{u,d}(q_d))(a)$, for some arbitrary arc $a \in F'$. Then, the equality ${{\,\mathrm{\textsf {reduced}}\,}}(c) = {\textsf {source}}_\beta$ implies that the property stated in (iv) is fulfilled. Finally, by the inductive assumption, we can compute $f_{u,d}(q_d), f'_{u,d}(q_d)$ in $\mathcal {O}(m^d)$ time. It follows that the functions $g_{u,d}(q_d), g'_{u,d}(q_d)$ can be computed in $\mathcal {O}(m^d)$ time. Hence we have shown (iii), (iv) and (v). $\square$

### Proof of Theorem 3

Consider the sink vertex *t*. By Lemma 9, the function $f_{t,d}$ has the property that some instance $q_d \in \mathbb {R}^{A_u^{(d)}}$ is linearizable iff $f_{t,d}(q_d) = 0$. For the proof of the theorem it is enough to show how to efficiently compute a matrix representation of $f_{t,d}$, i.e. a matrix $M\in \mathbb {R}^{k\times |A_u^{(d)}|}$ with $k = \mathcal {O}(m^d)$, such that $f_{t,d}(x) = Mx$ for all $x\in \mathbb {R}^{A_u^{(d)}}$. Then $\mathcal {L}_d$ is equal to the kernel of *M*, thus it can be computed in a time which is polynomial with respect to $|A_u^{(d)}|$ and *k*. Since $k =\mathcal {O}(m^d)$ and $|A_u^{(d)}| =\mathcal {O}(m^d)$ this computation time is polynomial in $\mathcal {O}(m^d)$, hence also polynomial with respect to the size of the input of the instance $q_d$ of the SPP_d_.

Now we turn to the efficient computation of *M*. Consider the set $e_1,\dots , e_k$ of all standard basis vectors of $\mathbb {R}^{A_u^{(d)}}$. Clearly, $k = \mathcal {O}(m^d)$. By Lemma 9, we can compute the vector $f_{t,d}(e_j)$ in $\mathcal {O}(m^d)$ time for every $j=1,\dots ,k$. Basic linear algebra tells us that these vectors constitute the columns of *M*. We can hence compute the matrix *M* and a basis of its kernel in $\mathcal {O}(m^{2d})$ time, which is polynomial with respect to the input of the $q_d$ instance of the SPP_d_. $\square$

## Conclusion

In this paper, we showed that the linearization problem for the QSPP on acyclic digraphs can be solved in time linear in the size of the input. Our algorithm significantly outperforms earlier algorithms and can also be generalized to the order-*d* linearization problem for all $d > 2$. The main insight behind our algorithm is a new characterization of linearizability, which reduces a global property to a local property. We showed that an instance of the generic shortest path problem (GSPP) on an acyclic digraph is linearizable if and only if every two-path system is linearizable. Hence it suffices to check all two-path systems for linearizability. Even though there is in general an exponential number of such systems, we introduced a polynomial-time algorithm which can check all of these systems simultaneously. Furthermore, we demonstrated how the runtime of the algorithm can be brought down to linear time using the idea of precomputed auxiliary values. We also showed how our ideas can be used to compute the linear subspace of all linearizable instances of the order-*d* SPP for a given fixed acyclic digraph. An anonymous referee pointed out that there might be a connection between the latter result and the Slater condition of a semidefinite programming formulation for the linearisation bound of the QSPP in the vein of the work of Hu and Sotirov [13]. This could be an interesting question subject to further research.

We remark that the linear runtime $\mathcal {O}(m^d)$ of our algorithm depends on the $\Omega (m^d)$ input cost coefficients to be dense (i.e. in the case $d=2$, they form a dense matrix). In the case of sparsely encoded input cost coefficients, it is an open question how to obtain a linear time algorithm.

Another open question related to the algorithm is its implementation for the practical relevant case of $d=2$, and the comparison of its perfomance on linearizable instances to a generic blackbox quadratic solver, which may not be aware that the input instance is linearizable.

At a theoretical level it would be interesting to investigate whether linearizable instances of SPP_d_ can be characterized by using reformulation–linearization techniques similar to those which have been successfully used for the characterization of linearizable QAP instances in [25].

## Footnotes

1 An extended abstract of this paper appeared in the proceedings of IPCO 2023 [4].

2 We use the same notation for the path *P* and the set of its arcs.

3 We assume that *f* is specified by an oracle.
